# ARES: Automated Risk Estimation in Smart Sensor Environments

**DOI:** 10.3390/s20164617

**Published:** 2020-08-17

**Authors:** Athanasios Dimitriadis, Jose Luis Flores, Boonserm Kulvatunyou, Nenad Ivezic, Ioannis Mavridis

**Affiliations:** 1 Department of Applied Informatics, University of Macedonia, 156 Egnatia Str., 54636 Thessaloniki, Greece; asdimitriadis@uom.edu.gr; 2 Industrial Cybersecurity, IKERLAN Technology Research Center, Basque Research and Technology Alliance (BRTA), P.J.M. Arizmendiarrieta 2, 20500 Arrasate/Mondragón, Spain; jlflores@ikerlan.es; 3 System Integration Division, National Institute of Standards and Technology, 100 Bureau Drive, Gaithersburg, MD 20899, USA; boonserm.kulvatunyou@nist.gov (B.K.); nenad.ivezic@nist.gov (N.I.)

**Keywords:** smart sensor environments, information system risk assessment, business process context, Common Security Standards

## Abstract

Industry 4.0 adoption demands integrability, interoperability, composability, and security. Currently, integrability, interoperability and composability are addressed by next-generation approaches for enterprise systems integration such as model-based standards, ontology, business process model life cycle management and the context of business processes. Security is addressed by conducting risk management as a first step. Nevertheless, security risks are very much influenced by the assets that the business processes are supported. To this end, this paper proposes an approach for automated risk estimation in smart sensor environments, called ARES, which integrates with the business process model life cycle management. To do so, ARES utilizes standards for platform, vulnerability, weakness, and attack pattern enumeration in conjunction with a well-known vulnerability scoring system. The applicability of ARES is demonstrated with an application example that concerns a typical case of a microSCADA controller and a prototype tool called Business Process Cataloging and Classification System. Moreover, a computer-aided procedure for mapping attack patterns-to-platforms is proposed, and evaluation results are discussed revealing few limitations.

## 1. Introduction

The fourth industrial revolution, commonly referred to as Industry 4.0, seeks to achieve self-aware, self-optimized manufacturing systems by applying service-oriented architecture to connect data analytics, digital contents, physical devices, and smart sensors across the supply network [1,2,3,4]. However, the adoption of Industry 4.0 presents many significant technical challenges [5], including integrability, interoperability, composability, and security [6,7,8]. Currently, integrability, interoperability and composability are addressed by next-generation approaches for enterprise systems integration such as model-based standards, ontology, Business Process Model Life Cycle Management (BPM LCM), context of business process, and a host of other transport protocol standards [9,10,11,12]. Security is addressed by conducting risk management as a first step.

According to the European Union Agency for Cybersecurity (ENISA), cybersecurity is necessary for Industry 4.0 adoptions [8]. The increased connectivity in Industry 4.0, the digitalization, and the advent of cloud services and sensors yield new cybersecurity risks. To achieve cybersecurity, organizations must perform risk management so that they can identify and access risks and keep them at acceptable levels. Risk management serves as a foundation on which organizations can start building a well-rounded cybersecurity strategy. The most fundamental component of risk management is risk assessment. Risk assessment has the purpose of identifying threats and calculating their risk levels allowing for the overall risk management to keep cybersecurity risks at acceptable levels [13,14]. According to the Risk Management Framework (RMF) proposed by the National Institute of Standards and Technology (NIST), risk assessment can be performed in three tiers: the organizational; business process; and Information Systems (IS) tier [14]. In addition, the NIST RMF emphasizes risk assessment automation in order that risks can be managed in a timely manner [14]. While risk assessment is important at all tiers, this paper proposes a novel approach about risk assessment at the IS tier (IS-related risk).

Numerous well-known risk assessment methodologies have been developed, proposed, and evaluated through different criteria [15], such as the OCTAVE [16] and MAGERIT [17]. Existing works, such as in [18], proposed the concept of business processes and the dependencies among them towards the estimation of the cascading risk. Other works, such as in [19,20], proposed the integration of the BPM LCM and risk assessment to enable risk automation. Additionally, Business Process Context (BPC), which is a set of meta-data about a business process model, was also specified as an important component in the BPM LCM that drives the risk assessment automation [13]. While these works pointed out the importance of BPM LCM and BPC in risk assessment, its automation via the BPM LCM and BPC is still sought.

In this paper, an approach for automated risk estimation in smart sensor environments, called ARES, is proposed. To enable automation of risk estimation, ARES integrates with BPM LCM. To that end, ARES exploits MITRE’s and NIST’s Common Security Standards (CSS) [21], the FIRST’s scoring system [22] and the tool called Business Process Cataloging and Classification System (BPCCS) developed in collaboration between the NIST and the Open Applications Group (OAGi) [3,10]. BPCCS provides a BPM LCM functionality to define the BPC in all the phases of modelling a business process (i.e., design, change, runtime). ARES takes advantage of the BPCCS for providing the BPC. As a result, changes in a business process cause a change in BPC that kicks off risk recalculation. Finally, ARES utilizes a proposed computer-aided attack patterns to platforms mapping procedure towards efficient risk estimation.

The rest of the paper is organized as follows: Section 2 presents the background necessary for presenting the ARES approach. Section 3 presents the related work concerning the utilization of MITRE’s common security standards to identify threats and assess IS-related risk, as well as the integration of risk assessment with the business process management. Section 4 describes the proposed ARES approach. Section 5 presents an application of the ARES approach utilizing the BPCCS. In Section 6, the proposed computer-aided attack patterns-to-platforms mapping procedure is presented. Section 7 discusses the results and the limitations found, and Section 8 concludes the paper.

## 2. Background

This section describes concepts necessary for subsequent discussions and for enabling ARES.

### 2.1. Risk Assessment

A risk is a measure of the extent to which an entity is threatened by an event [23]. Risk should be kept at acceptable levels to justify the security of a system [14]. To do so, risk management is conducted. Risk management aims to identify risks, assess risks, and keep risks at acceptable levels [13,14]. All in all, risk management serves as a foundation on which organizations can start building a well-rounded cybersecurity strategy [13].

**Figure 1:**
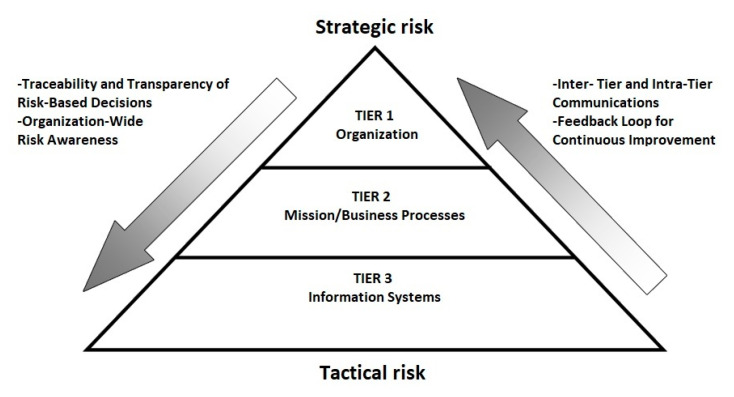
Multitiered risk management and risk assessment from [14].

Risk management is composed of four components: framing the risk (i.e., establish the context for risk-based decisions), assessing the risk, responding to the risk, and monitoring and control the risk [14,23]. The most fundamental component is assessing the risk (i.e., risk assessment or risk estimation) [23,24]. The purpose of risk assessment is to estimate the extent to which an entity is threatened by a potential circumstance or event, namely, to estimate the risk [14,23,24]. The importance of automating risk assessment has been pointed out in the literature since it leads to a well-built risk management plan and timely decision-making [14]. According to NIST, both risk management and risk assessment are conducted at three tiers: (i) organization; (ii) mission/business process; and (iii) information systems tier, as depicted in Figure 1 [14,23].

Although risk assessment at all tiers is important, this effort focuses on risk assessment at the IS tier. According to NIST’s guides [24], a risk assessment methodology includes:

(1) A risk model, which defines the risk factors, their relationships used in calculating the level of risk. Some examples of risk factors include threats, vulnerabilities, the impact of a threat, the likelihood of a threat, and predisposing conditions. In order for a risk model to use these risk factors, they should be estimated first. To estimate the risk factors, different approaches have been proposed, such as using the rate of occurrences of a threat [17] or formal distributions [25] to estimate the likelihood of a threat.
(2) An analysis approach, which defines how the risk factors are identified and analyzed. An analysis approach can be asset/impact-oriented, threat-oriented, or vulnerability-oriented. The main difference between analysis approaches is their starting point. An asset/impact-oriented approach starts with the identification of impacts and critical assets. A threat-oriented approach starts with the identification of threat sources or threat events. On the other hand, a vulnerability-oriented approach begins with the identification of exploitable weaknesses in the information systems. However, all of them should first identify the assets that need protection. In a threat-oriented approach, there is no way to identify threats without knowing the assets that need protection against them, neither in a vulnerability-oriented approach nor in an asset-oriented.
(3) An assessment approach, which specifies the range of the values of the risk. An assessment approach can be quantitative, qualitative, or semi-qualitative. A quantitative approach uses numbers, a qualitative approach uses non-numerical categories or levels (e.g., very low, low, moderate, high, very high), and a semi-qualitative uses predefined value scales (e.g., 0–5, 7–8 or 8–10).
(4) A risk assessment process, which includes the activities for preparing the risk assessment, conducting risk assessment, communicating the results and maintaining the assessment. The risk assessment is conducted using an analysis approach to identify the risk factors and to evaluate them, and a risk model is to estimate risks according to an assessment approach.

### 2.2. Common Security Standards

**Figure 2:**
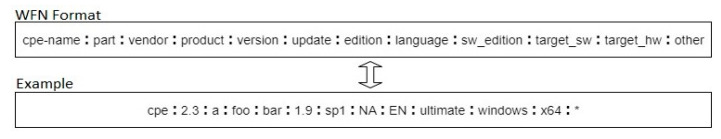
Well-formed CPE name structure.

Targeted at both the software development and security practitioner communities, the following MITRE’s and NIST’s Common Security Standards [21], as formal lists or dictionaries supporting security content automation, are utilized in this paper:

(1) Common Platform Enumeration (CPE) Scheme [26,27,28]: CPE is an abstract structured naming scheme for describing and identifying applications, operating systems, software, and hardware, including industrial control systems, such as supervisory control and data acquisition (SCADA) The logical construct of a CPE is called “Well-Formed CPE Name (WFN)”. Figure 2 depicts the WFN format and provides an example of a CPE [28].
  The CPE scheme, however, cannot describe and identify specific instances of products (e.g., using serial numbers, particular licenses, or physically discernible products) [28]. For instance, CPE can describe the XYZ Visualizer Enterprise Suite 4.2.3, XYZ Visualizer Enterprise Suite (all versions), or XYZ Visualizer (all variations) but not the XYZ Visualizer Enterprise Suite 4.2.3 with the serial number Q472B987P113 [28].
(2) Common Vulnerabilities and Exposures (CVE) [29,30]: A CVE is a known weakness found in specific software or hardware that can be exploited and result in an impact on Confidentiality, Integrity or Availability (CIA) attributes. Each CVE includes a unique identification number, a description, one public reference, a reference to the software or hardware that is affected (using CPE), a reference to a weakness (described in the next bullet), and its severity (described in Section 2.3).
(3) Common Weakness Enumeration (CWE) [31]: CWEs represent flaws in software architecture, design, or code before fielding that can lead to a specific CVE in an application. A CWE list is a set of weaknesses, each associated with some CVEs. For example, CVE-2019-1675 “Cisco Aironet Active Sensor Static Credentials Vulnerability” existing in the Cisco Aironet active sensor is a manifestation of the CWE-798 “Use of Hard-coded Credentials”. Therefore, it can be assumed that CVEs are “known knowns” (things we are aware of and understand) or specific vulnerabilities, while CWEs are “unknown knowns” (things we understand but are not aware of) or generic vulnerability types.
(4) Common Attack Pattern Enumeration and Classification (CAPEC) [32]: CAPEC is a comprehensive dictionary and classification taxonomy of known security threats. Each CAPEC describes the common characteristics of a cyber threat (aka attack-pattern), helping to explain how applications and other cyber-enabled capabilities are likely to be attacked. CAPEC also provides the likelihood that the attack pattern can occur and its impact. CAPEC utilizes a qualitative approach rating both likelihood and impact in a five-step value scale ranging from very low to very high. Finally, each CAPEC records the weaknesses (CWEs) that the attack pattern can exploit.

### 2.3. Common Vulnerability Scoring System

The Common Vulnerability Scoring System (CVSS), which was developed by the Forum of Incident Response and Security Teams (FIRST), is a framework for communicating the characteristics and severity of CVEs [22]. CVSS represents the severity of a CVE using a summarized score, and the following three metric groups [22]:

(1) The Base Metrics that reflect the intrinsic characteristics of a CVE. These characteristics are constant, and they are categorized into the following two groups:
  (a) The exploitability metrics that represent “the ease and technical means by which the vulnerability can be exploited”, and
  (b) The impact metrics that represent the impact of the CVE on each CIA attribute. The values of the impact metrics can be “None”, “Low” or “High”, depending on whether there is no loss, some loss, or total loss of the CIA attribute.
(2) The Temporal Metrics that adjust the Base Metrics based on factors that can change over time, such as the availability of exploits that take advantage of the CVE.
(3) The Environmental Metrics that adjust the Base and Temporal Metrics to a specific environment considering more specific factors such as the presence of mitigations (countermeasures) that exist in that environment.

**Figure 3:**
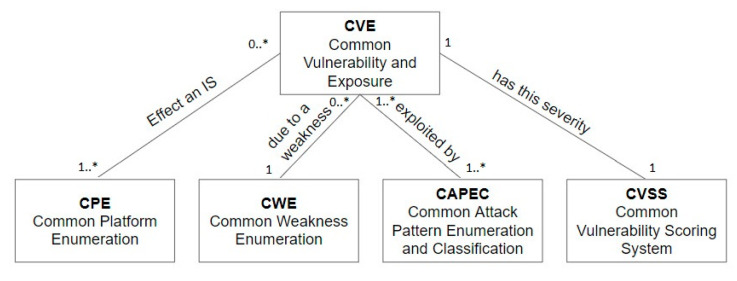
Relationships among CSSs and CVSS.

The UML diagram of Figure 3 depicts the relationships among the common security standards and CVSS. As the diagram depicts, a CVE has a CVSS severity, effects a CPE due to a CWE, and it is exploited by a CAPEC.

**Figure 4:**
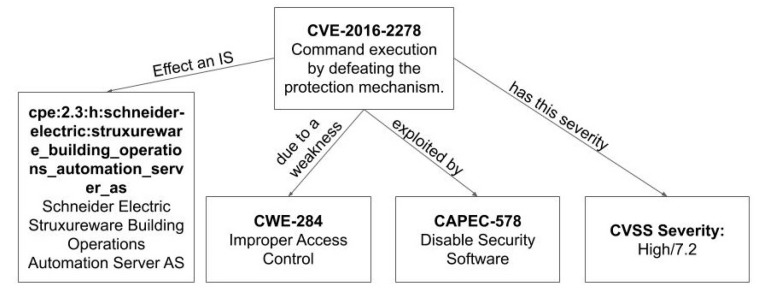
Application example of relationships among CSSs and CVSS.

Figure 4 presents an example of relationships among CSSs and CVSS in a real case related to the CVE-2016-2278 that effects the platform “Schneider Electric StruxureWare Building Operations Automation Server AS” due to the weakness CWE-284. The particular CVE has a CVSS severity High or 7.2, and it is exploited by applying the attack pattern CAPEC-578.

### 2.4. Business Process Cataloging and Classification System

**Figure 5:**
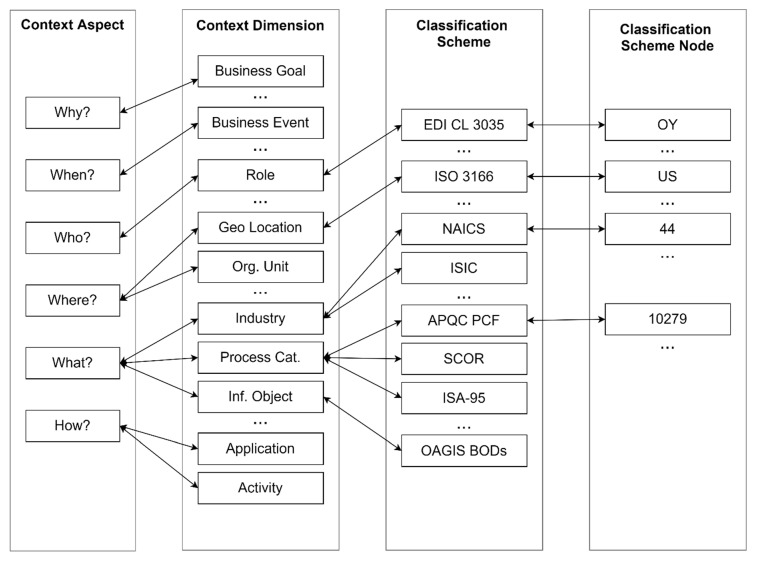
Application example of BPC definition from [10].

The BPCCS is an innovative prototype tool, being developed by NIST in collaboration with the OAGi, whose aim is to increase the productivity of standards-based integration through the use of BPC [3,10]. The BPCCS relies on BPM LCM and allows for defining the BPC of a business process using the context aspects—Why, When, Who, Where, What and How (5 WH)—derived from the Zachman’s framework and its introduction of the 5 WH maxims, as depicted in Figure 5 [10]. Each Context Aspect consists of multiple Context Dimensions that their purposes are related to the corresponding context aspect. Each Context Dimension can have a value, whose domain is defined using one or more Classification Schemes. The selected values of each Context Dimension are called Classification Scheme Nodes. Finally, the set of the Classification Scheme Nodes forms the BPC.

**Figure 6:**
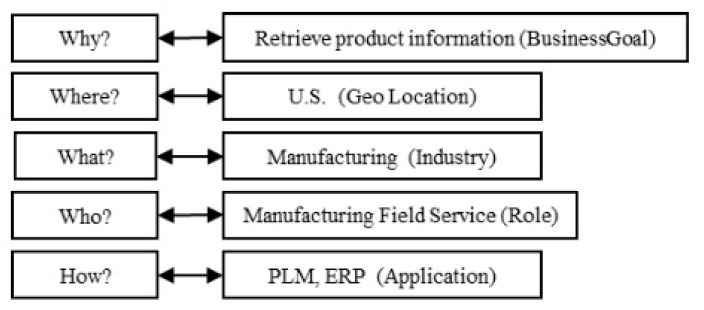
The BPC for the “Retrieve ECU Information Sub-process” from [10].

Figure 6 depicts an example of a BPC as a set of Classification Scheme Nodes for the business process “Retrieve Electrical Control Unit (ECU) Information Sub-process” from a business scenario where a manufacturing plant production line has had a breakdown [11].

## 3. Related Work

This section reviews two bodies of related work found in the literature that address concerns targeted by this research. The first body of work is concerned with the utilization of MITRE’s CSS to identify threats and assess IS-related risk. The second body of work dealt with the integration of risk assessment with business process management.

### 3.1. Utilizing MITRE’s Common Security Standards to Identify Threats and Assess IS-Related Risk

The Knowledge Graph-Based Intelligent Alert Correlation Framework (KGBIAC) allows alert correlation analysis to be conducted by utilizing knowledge graphs to represent alerts and network information [33]. Alerts come from security tools, such as firewalls and intrusion detection systems (e.g., Snort), while network security information (e.g., vulnerabilities) comes from the systems that operate inside the networks. To correlate all the information and synthesize graphs, the system normalizes the alerts while using structural representation languages for assets and vulnerabilities, such as CPE, CVE, and CWE. Moreover, the KGBIAC uses the CAPEC schema to represent the attack patterns related to an alert. After fusing all this information, a graph is synthesized to be used for analyzing network security alert correlation. Although KGBIAC identifies alerts and, therefore, it can identify threats, it does not focus on risk assessment.

In [34], authors proposed a technique and a software tool for generating cyber-attack scenarios backward and, therefore, to identify threats in such a way. The tool randomly picks an attack pattern from CAPEC and uses its pre- and post-conditions fields, along with CVE, CVSS, and CPE, with the goal of finding a host in the network to gain access. Afterward, it analyses the impact of the attack on the host in terms of CIA attributes. This is a black-box approach since there is no prior knowledge of the network and hosts operating inside it. Although this approach uses common security standards, it cannot be used for risk assessment. Finally, the work claimed that the environment and platform fields of CAPEC were used to choose a CPE that was most closely related to the selected CAPEC. Based on our research, however, it is discovered that these fields are seldom used. This is described further in Section 7.

In [35], a risk prediction methodology based on historical data about CVEs and CVSS was introduced with the following steps: (1) platform description using CPE; (2) identification of previous vulnerabilities of the platform by retrieving and examining historical data from the National Vulnerability Database (NVD) in the form of CVEs (each CPE can be related to multiple CVEs); (3) vulnerability prediction by applying Kolmogorov-Smirnov tests to reveal whether the number of future vulnerability occurrences will be less or greater; (4) risk prediction by utilizing a risk model based on a Bayesian Belief Network (BBN) topology, Von Mises’ theory, and predicted future CVEs (step 3) and their CVSS. The methodology uses historical data to predict the trend of the number of vulnerabilities into the future. The prediction is only from the vulnerability perspective without considering threats (e.g., CAPEC). However, threats must also be identified and be taken into consideration when the risk is estimated [24]. Furthermore, the CVSS impact values on CIA attributes may be higher compared to the impact that a CAPEC can cause. Without considering CAPEC, the above methodology may overestimate risk. This situation is described in further detail in Section 4.

In [36], a risk assessment method for banking IS was proposed. The method assesses the risk of a banking IS utilizing the standard security requirements of banking (SSRB) extracted from best practices of the banking sector, and the CAPEC. The authors proposed that the risk of a banking IS is the sum of the risks of the missing SSRBs of the banking IS being examined. To assess the risk of each SSRB, SSRBs are mapped to CAPECs, and then the risk of each SSRB was deemed to be the sum of the risks of the related CAPECs. To identify the missing SSRBs of a banking IS, text-similarity techniques, such as the Frequency-Inverse Document Frequency weight, are used to map SSRBs to the security requirements document of a banking IS. Any SSRB deemed unmatched was considered to be a missing requirement, and therefore, its risk is used to calculate the overall risk. While the method provides quantitative risks and the assessment can be conducted fast and based on banking sector standards, it is not based on a specific risk model (e.g., likelihood, impact, threat, vulnerabilities) such that it can reflect risk based on the existing vulnerabilities of the IS and their threats. Moreover, the method is limited to utilizing only CAPEC.

### 3.2. Integrating Risk and Business Process Management

In [37,38,39], the authors proposed a methodology for enabling risk-aware business process management and a simulation called Risk-Oriented Process Evaluation (ROPE). A core concept of this approach is the process-oriented modelling of threats and detection, countermeasures and recovery measures for each threat. The ROPE methodology consists of five iterative steps and a three-modelling layer in order to identify threats and their management and recovery strategy. To do so, the modelling layer starts with identifying the crucial business processes and their activities for prioritization purposes regarding the detection of threats. At the next layer, the discovery of the ISs of the activities of the business processes takes place. Finally, at the third layer, the threats and their management and recovery strategy are identified. Although ROPE methodology identifies threats by utilizing business processes, it does not utilize the common security standards to describe the ISs in a structured way. Another critical component that ROPE lacks is a risk assessment model used for estimating risk. Without the above mentioned, neither risk assessment can be supported nor can be automated.

In [40], a multi-layer integration of business process management and risk management was proposed, focusing on the design phase of business processes. The authors integrated the conceptual model of risk management from their previous work [20] with additional concepts related to business processes to create a single conceptual model. Additionally, a method that synchronizes the life cycles of risk and business process management was used to support the operational deployment of their conceptual model. Although their proposed synchronized life-cycle management method provides guidance to conduct risk management during the design phase of a business process, it only provides high-level detail regarding risk assessment.

In [41], a proactive risk management approach during a business process execution was proposed. The approach consists of three parts. First, an approach for analyzing the behavioral requirement of a proactive process-related risk management was proposed. It specified necessary activities to handle risks. Second, a framework for monitoring and treating risks during the runtime of a business process was provided. The last part furnished the framework with a specialized rule language. The overall approach was shown to handle not only negative impacts generated by risks but also the positive ones (a positive impact can be referred to as an opportunity). However, the effort was focused on the exceptions that might occur during the runtime of a business process and how to handle them rather than on threats/attacks (e.g., attack patterns described by CAPEC).

## 4. The Proposed ARES Approach

Business continuity and risk management experts suggest that the risk assessment should be conducted within the context of a business process [40]. According to the NIST CyberSecurity Framework, “understanding the business context, the resources that support critical functions, and the related cybersecurity risks enables an organization to focus and prioritize its efforts, consistent with its risk management strategy and business needs” [13]. Our proposed approach aims at utilizing the BPC and the CSSs in order to provide an automatic assessment of IS risk of business processes.

The ARES approach complies with the NIST SP 800-37 guidance for conducting risk assessment as outlined in Section 2.1. ARES follows a qualitative approach to estimate risk for each CIA attribute of a CPE. The risk estimation of ARES is based on a risk model that is a product of the likelihood of a threat (derived from a CAPEC) occurring and the potential impact incurred by this threat on each CIA attribute. The product of the likelihood and the impact results in the following range of values: None, Low, Medium, High, and Very High, as shown in Table 1.

**Figure 7:**
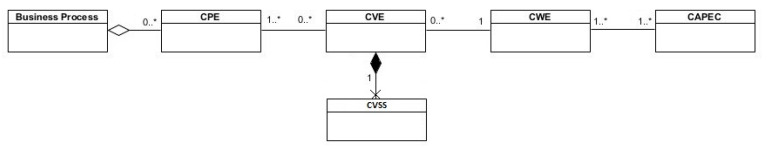
Class diagram of the vulnerability-oriented analysis approach of ARES.

To identify the risk factors of the risk model, namely the likelihood and impact, ARES follows a vulnerability-oriented analysis approach. Specifically, this vulnerability-oriented analysis approach utilizes the relationships defined in the UML class diagram of Figure 7. This figure illustrates that a business process might be supported by multiple CPEs (assets). Each CPE can have none or multiple vulnerabilities (CVE). A CVE includes a CVSS score and can be associated with a weakness (CWE) that may be exploited by at least one CAPEC.

**Figure 8:**
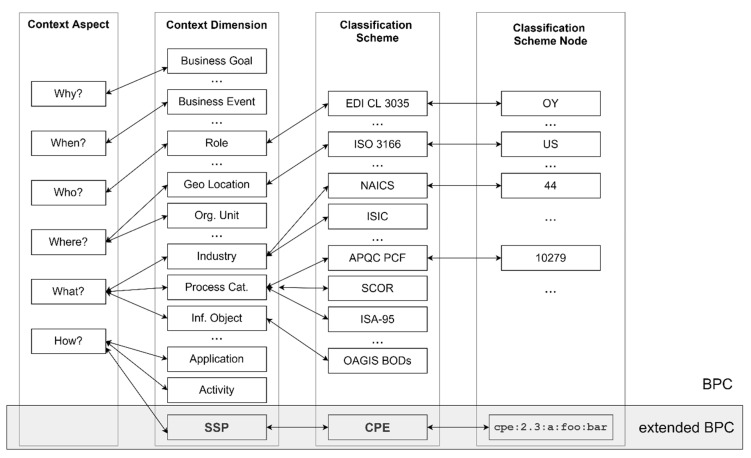
The proposed extension of the Business Process Context based on [10].

As mentioned in Section 2.1, the vulnerability-oriented analysis approach should first identify the assets that need protection—the CPEs supporting the business process. To enable the discovery of CPEs, we extended the BPC, as defined by the BPCCS (Figure 8). In particular, we propose the Context Aspect “How” to include the Context Dimension “Systems, Software, and Packages” (SSP in Figure 8) using entries from “Common Platform Enumeration” (CPE) as the classification scheme.

ARES estimates the risk for each CIA attribute of a CPE based on the impact and likelihood of the related CAPECs. However, a CPE might have multiple CVEs, each of which may be exploited by multiple CAPECs (Figure 7). Therefore, each CIA attribute might face multiple risks. We propose that the overall risk for each CIA attribute of a CPE is not based on the sum of all risks. Rather, an upper bound risk based on the worst-scenario CAPEC is used. This approach of upper bound risk supports risk aggregation of several risks of lower tiers (e.g., the IS tier) into a general risk of the upper level (e.g., the business process or organization tier); a notion suggested by NIST [24]. The worst-scenario CAPEC is the CAPEC with the highest likelihood and impact values. ARES also validates that the worst-scenario CAPEC can really pose a threat using a CAPECs-to-CPE mapping. If the worst-scenario CAPEC cannot pose a threat to the CPE, the next worst-scenario CAPEC is selected and validated again.

In this research, we propose a computer-aided approach to create a CAPECs-to-CPE mapping using a biclustering technique. The biclustering techniques have been used in the analysis of DNA microarrays for finding possible relationships between genes (in our case, CPEs) and diseases (in our case, CAPECs) [42,43]. The approach is detailed in Section 6.

For the likelihood factor, ARES uses the likelihood of the worst-scenario CAPEC as is. For the impact factor, ARES compares the impact value of the worst-scenario CAPEC with the CVSS impact value of the CVE that this CAPEC can exploit. The impact factor is the lowest value, as shown in Table 2. If the CAPEC does not provide an impact value, ARES uses the impact value of its parent CAPEC. In the case that CVSS does not provide impact value, ARES uses the CAPEC’s impact value. Overall, we propose that the impact factor estimation should depend on the CVSS, since the CVSS can be the bottleneck of the impact of a CAPEC on a CIA attribute. This value estimation approach complies with MITRE’s statement that the impact of a CAPEC can vary depending on the particular context of the target software under attack [44]. In other words, the impact should be estimated by comparing the impact of a CAPEC to the impact that a CVE can cause.

After the impact and the likelihood factors have been estimated, the risk for each CIA attribute can be determined based on their product according to Table 1.

**Figure 9:**
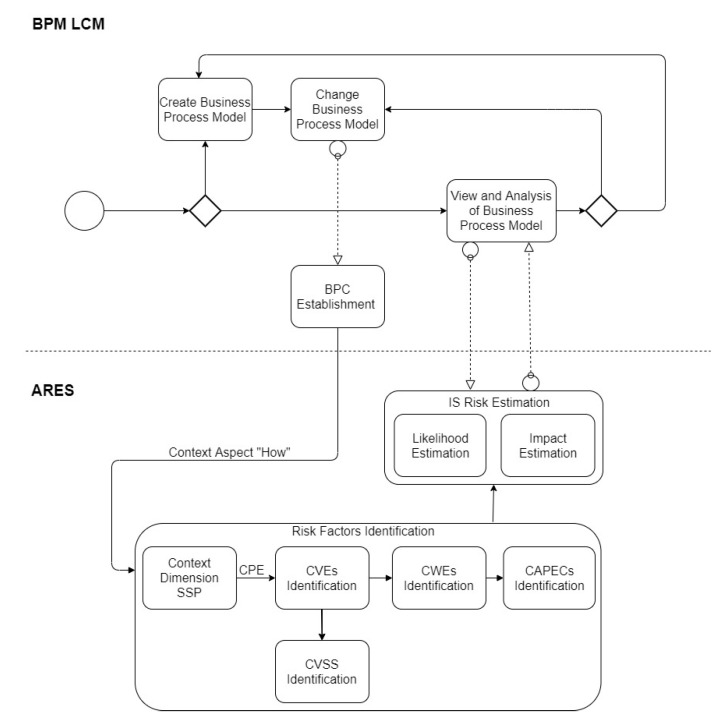
The proposed integration of BPM LCM and ARES.

To enable risk recalculation in every BPC change, we propose an integration of BPM LCM and ARES based on the notion of [40] where the life cycles of business process and risk management are integrated. Figure 9 depicts how the automation works. It shows that whenever a business process model changes (the “Change Business Process Model” box), the BPC gets updated (the “BPC Establishment” box) in the BPM LCM layer that triggers all the risk estimation activities on ARES layer. This is described in more detail in the next section with an example.

## 5. Utilizing BPCCS in Applying the ARES Approach

**Figure 10:**
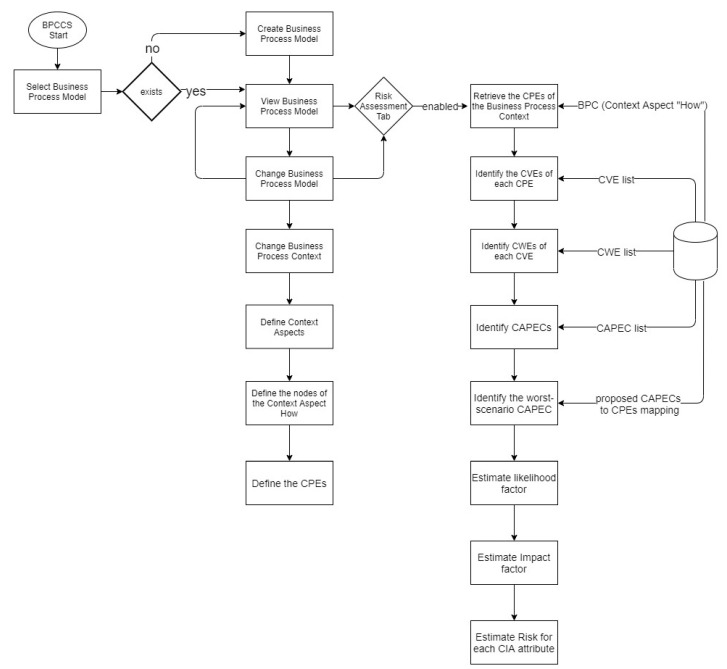
Flowchart diagram of utilizing BPCCS in applying the ARES approach.

The flowchart diagram shown in Figure 10 depicts the utilization of BPCCS in applying the ARES approach. The risk is estimated and presented for each CIA attribute of each CPE supporting the business process under consideration.

Whenever a business process model is created or changed, its BPC should be defined or changed accordingly. A BPC can be changed by changing the classification scheme nodes of each of 5WH context aspects. The context aspect used by ARES is the context aspect “How”. As proposed in Section 4, the context aspect “How” documents the CPEs supporting a business process.

Having the CPEs defined under the context aspect “How”, the BPCCS can retrieve respective CVEs and CWEs provided by the NVD of NIST [29]. It can then retrieve the CAPECs that may be able to exploit those CWEs using MITRE’s database. An illustrative example of the result of the above-mentioned process is shown in Table 3. In particular, Table 3 shows the IDs of the CVE, CWE, and CAPEC for the CPE “cpe:2.3:a:abb:microscada_pro_sys600:9.3:*:*:*:*:*:*:*” (i.e., ABB MicroSCADA Pro SYS600 9.3; a controller that gathers information from network sensors, and provides an interface to operators for their further actions). For instance, the ABB MicroSCADA Pro SYS600 9.3 has the CVE 2019-5620 that is a manifestation of CWE-306 (i.e., “Missing Authentication for Critical Function”), which can be exploited by CAPEC-36 (i.e., “Using Unpublished APIs”). In other words, an attacker can gain access to the ABB MicroSCADA Pro SYS600 9.3 by exploiting its API that fails to authenticate requests.

The next step is to select the worst-scenario CAPEC. To do so, the likelihood and impact of the CAPEC-12, CAPEC-36, CAPEC-62 and CAPEC-166 are compared. The worst-scenario CAPEC is CAPEC-62, since it has the highest impact and likelihood values (i.e., High and Very High). To verify that the CAPEC-62 can indeed pose a threat to the “cpe:2.3:a:abb:microscada_pro_sys600:9.3:*:*:*:*:*:*:*”, the proposed CAPECs-to-CPEs mapping procedure is utilized. As further explained in Section 6, the CAPECs-to-CPEs mapping is a result of applying biclustering techniques to create an XML file containing for each one CAPEC all possible associated CPEs. This XML file is used for validating that the worst-scenario CAPEC (in this example CAPEC-62) can pose a threat to the associated CPE being examined (in this example the microSCADA controller). According to the CAPECs-to-CPEs mapping, only CAPEC-36 is associated with that CPE; therefore, no other CAPECs can pose a threat to the “cpe:2.3:a:abb:microscada_pro_sys600:9.3:*:*:*:*:*:*:*”. For instance, CAPEC-62 (Cross Site Request Forgery) cannot even be applied to the ABB MicroSCADA Pro SYS600 9.3, since it can only affect websites. Because of this, the worst-scenario CAPEC in the above example is the CAPEC-36.

Afterward, the likelihood and impact factors are estimated. The likelihood risk factor is set to the likelihood value that CAPEC-36 provides. In our case, the value of the likelihood risk factor is “Medium”. The impact risk factor is estimated by using the lowest value between the impact value of the CAPEC-36 and CVSS of the CVE 2019-5620 (each CVE contains its CVSS score). The impact value of CAPEC-36 is High, and the impact value of the CVSS is High for each CIA attribute. Therefore, the impact factor of the CAPEC-36 is High for all CIA attributes. 

Finally, the risk is estimated by following the risk model of the ARES shown in Table 1. The results are presented for each CIA attribute in Table 4.

## 6. Attack Patterns-To-Platforms Mapping

In this section, a computer-aided procedure to map attack patterns (CAPECs) to platforms (CPEs) using biclustering is proposed. The purpose of CAPECs-to-CPEs mapping is to validate that the worst-scenario CAPEC can pose a threat to the CPE being examined.

In order to accomplish the proposed mapping, we used part of the CPE list (about 40,000 CPEs out of 400,000 total). To each CPE, a software type (CPE category) was assigned using the proposed categories of [45]. For instance, we assigned the CPE category “compression_decompression” to all “7zip” versions, the CPE category “email” to all “Thunderbird” and “Outlook” versions, “word_processing” to all “Microsoft Word” versions, “web_browsers” to all “Google Chrome” and “Mozilla Firefox” versions and so forth. The purpose of assigning CPEs categories is to support the validation of the mapping at the end of the procedure. Afterward, ARES was applied to identify the threats (CAPECs) that each CPE might face utilizing its analysis approach (Figure 7). The result of threat identification was a list with all CAPECs that a CPE might face based on the historical data utilizing the existing CVE lists.

**Figure 11:**
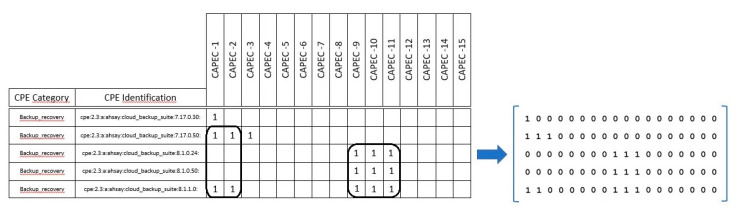
Example of locating associations among CAPECs and CPEs.

This list was represented as a binary matrix where the rows were the CPEs, and the columns were the CAPECs. Each cell represented the presence or absence of a specific CAPEC in a specific CPE, as depicted in Figure 11.

The CAPECs that are likely to pose real threats to CPEs are the CAPECs that are associated with the same CPEs, and when those CPEs also have the same CAPECs. That is, for a specifically selected group of CPEs, the CAPECs should be the same and vice versa. This requirement is translated to the biclustering problem as follows.

Based on the matrix (left of Figure 11), a binary matrix was created (right of Figure 11) in which the rows represent the CPEs, the columns represent the CAPECs, and each cell represents the presence (1) or absence (0) of the CAPEC associated with the CPE. The goal was to find the maximum submatrices with all 1′s representing the maximum number of CPEs with the same CAPECs. This problem is known as a biclustering problem. This kind of problem has been widely studied in the analysis of DNA microarrays for finding relationships between genes (in our case, CPEs) and diseases (in our case, CAPECs) [42,43].

The goal of applying biclustering techniques is to identify groups of CPEs and groups of CAPECs, by performing simultaneous clustering of both rows and columns of the CAPEC to CPE binary matrix instead of clustering these two dimensions separately. To illustrate, let us consider the general case of a binary data matrix, A, with a set of rows, X, and a set of columns, Y, where the element $a_{ij}$ describes the relationship between row i and column j in terms of presence or absence. This matrix can be defined by the set of rows, $X=\left\{x_{1},\ldots ,x_{n}\right\}$, and the set of columns, $Y=\left\{y_{1},\ldots ,y_{m}\right\}$, where (X,Y) denotes the matrix A. Based on this definition, we can consider I⊆X, J⊆Y as the subsets of rows and columns, respectively. $b_{IJ}=\left(I,J\right)$ denotes the submatrix (i.e., bicluster) of the matrix A with the set of rows I and the set of columns J. A bicluster is a subset of rows that are similar to a subset of columns and vice versa as measured by a coherence value. The coherence value of a subset is determined by utilizing the Univariate Marginal Distribution Algorithm (UMDA) as it is further described in [46,47]. In our case, the coherence of a bicluster (submatrix of CAPECs and CPEs) is higher when all CAPECs have been selected for all CPEs, that is, the matrix only contains “1 s”, and the coherence is the minimum when the matrix only contains “0 s”. Based on this definition, it is possible to search for the best biclusters, that is, the best submatrices of CAPECs and CPEs. A bicluster $b_{IJ}=\left(I,J\right)$ is thus a subset of rows and a subset of columns where $I=\left\{x_{1},\ldots ,x_{k}\right\}$ is a subset of rows (I⊆X and k≤n), and $J=\left\{y_{1},\ldots ,y_{s}\right\}$ is a subset of columns (J⊆Y and s≤m), viz a bicluster can be defined as a k by s submatrix of matrix A. Now, it is possible to define the problem addressed by our biclustering algorithm. Given a data matrix, A, identify a set of biclusters B whose member is a bicluster $b_{I_{z}J_{z}}=\left(I_{z},J_{z}\right)$ such that B optimizes the coherence [48,49].

To identify the coherent biclusters, there are various biclustering approaches in the literature. In our study, a conservative, iterative approach, which has been demonstrated to be very effective in the field of genetic data analysis was adopted. It uses a merit function to measure the coherence of a candidate submatrix of CAPECs and CPEs. The merit function is based on a heuristic optimization paradigm known as the Estimation of Distribution Algorithms (EDA) [46,50]. The paradigm of EDAs is a set of stochastic optimization algorithms that can guide the search for the optimum coherence value by sampling and estimating the parameters of an explicit probabilistic model of a set of solutions that represent different points of the model [49]. The model we chose is known as Univariate Marginal Distribution Algorithm for discrete domains (UMDA), and is analyzed in [46,49].

The optimization is an iterative process where the parameters are improved until the optimum value is obtained; the optimum value is one that fulfills the conditions of the biclustering problem. Initially, a random sample of candidate data (the initial population), R, is generated [46]. These candidates are evaluated using an objective function and based on this evaluation, the optimum points are selected. Afterward, a probabilistic model of the selected solutions is learned and used to sample a new set of points. This process is iterated until the optimum is found or a termination criterion is reached [46].

The use of the UMDA for mapping CAPECs to CPEs assumes that all variables are independent, and the factorization of the joint probability is computed as the product of univariate marginal probabilities. Indeed, the variables of our biclustering problem is a set of independent Bernouilli variables where ‘0’ represents the absence of an association between a CAPEC and a CPE, and the ‘1’ represents the presence. The joint probability can be learnt as follows [46]:

(1) $$p_{l}\left(x\right)=\prod_{i=0}^{n}p_{l}\left(x_{i}\right),$$

This distribution represents the product of *n* independent probability distributions associated with the Boolean input and output configurations. $p_{l}\left(x_{i}\right)$ is a Bernoulli probability distribution of the *l-*th iteration that takes two values, the possible Boolean values ‘0’ and ‘1’. The estimation of the parameters of the model is performed based on marginal frequencies of the selected subset of the previous generation (i.e., $D_{l-1}^{S_{e}}$) [46]:

(2) $$p_{l}\left(x\right)=\frac{\sum_{i=1}^{n}\delta_{j}(X_{i}=x_{i}|D_{l-1}^{S_{e}})}{R}$$

where:

(3) $$\delta_{j}\left(X_{i}=x_{i}|D_{l-1}^{S_{e}}\right)=\{\begin{array}{c} 1,\;\mathrm{if}\;\mathrm{in}\;\mathrm{the}\;l-\mathrm{th}\;\mathrm{case}\;\mathrm{of}\;D_{l-1}^{S_{e}},X_{i}=x_{1}\\ 0,\;\mathrm{otherwise} \end{array},$$

**Figure 12:**
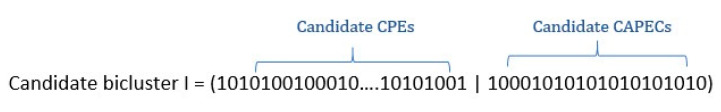
Candidate bicluster.

**Figure 13:**
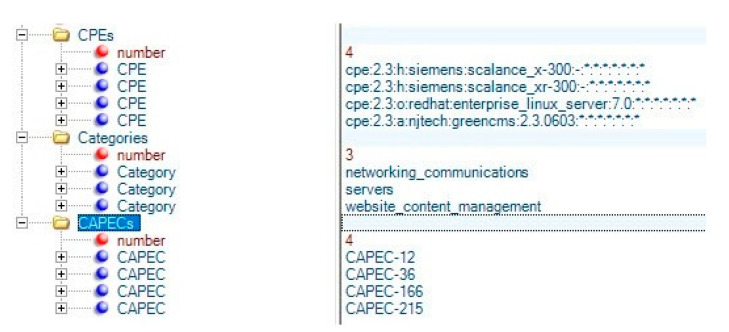
Example of a subgroup of proposed CAPECs to CPE mapping.

A new population is obtained by sampling the built joint probability distribution from selected candidate solutions. A complete candidate solution (*X*) will consider all possible input values represented by a binary vector. In our case, the binary vector encodes the selection of candidate CPEs and CAPECs to be evaluated as depicted in Figure 12:

The algorithm evaluates the coherence of this selection and, according to the generic pseudocode will be selected for learning (estimating the Bernouilli parameters) or rejected until the population reaches the optimum value. The final candidate will represent the optimum bicluster, that is, the most coherent subgroup of CAPECs and CPEs. Our biclustering algorithm is an iterative algorithm that receives as input the matrix of the CPEs and CAPECs and utilizes the UMDA to extract a list of the optimum biclusters (CAPEC-CPE subgroups). The CAPECs of each optimum bicluster discovered are removed from the matrix, and the UMDA is used again to find the next bicluster until all CAPECs are used/found. The final result can be represented as an XML file containing the subgroups of CPEs, their CPE categories, and their relevant CAPECs. An illustrative example of such subgroup is depicted in Figure 13.

In the last step, biclustering results are verified by a human to whether the CAPECs in the subgroup can pose threats to the CPE. It is done so by verifying the CAPEC description against the CPE category. Whenever there was a false positive CAPEC (a CAPEC that cannot pose threats to the CPE category), another subgroup was created with the CAPEC and the CPE categories that can be affected by the CAPEC.

The resulting XML file is used to ensure that the selected worst-scenario CAPEC can pose a threat to the CPE category and, in particular, to the CPE being examined. In this way, it can be assured that the risk is calculated based on a CAPEC that not only is the worst-scenario but also is applicable to the CPE being examined.

## 7. Discussion

Using ARES, risk can be assessed in smart sensor environments in an automatic manner. The integration of BPM LCM with ARES allows risks to reflect changes to BPC, continuously and in real-time. ARES can be applied in various environments, from small- and medium-sized enterprise environments to industrial ones where different kinds of information systems, sensors and sensor-based platforms are operated.

In order to evaluate and to validate the applicability of ARES and document any potential limitations, the BPCCS was utilized. During this evaluation, two limitations were discovered. The first limitation is that ARES cannot be used in estimating the risk of CPEs defined in the CPE list of 2007. This list does not include the CWEs of the CVEs: Each CVE provides the generic value “NVD-CWE-Other” in its CWE field. The second limitation is that an identified CAPEC might not pose a threat to the CPE being examined, leading to an incorrect risk calculation. For instance, when we applied ARES, we realized that the CPE “cpe:2.3:a:abb:microscada_pro_sys600:9.3:*:*:*:*:*:*:*” (i.e., ABB MicroSCADA Pro SYS600 9.3) has the CVE 2019-5620, which is a manifestation of CWE-306 (Missing Authentication for Critical Function). According to CAPEC data, CWE-306 can be exploited by CAPEC-62 (Cross Site Request Forgery). However, CAPEC-62 is an attack pattern that is related to websites; therefore, it cannot affect a microSCADA controller, such as the “ABB MicroSCADA Pro SYS600 9.3”. In this case, if the CAPEC-62 was the worst-scenario CAPEC, then the risk estimation would be inaccurate. This issue was overcome with the proposed attack patterns to platforms mapping. The CAPEC, which is selected, must not only be the worst-scenario CAPEC but must also pose threat to the CPE being examined. To ensure the threat posture, a validation is needed. While the validation can be done entirely manually; this paper proposes a computer-aided approach to speed up the process.

Compared to the ARES approach, no other risk assessment methodology offers such automation in conducting risk assessment utilizing the BPC. Although there are efforts synchronizing the BPM LCM and risk assessment such as in [40], they provided only high-level details regarding risk assessment automation. In contrast, the ARES approach adds adequate granularity needed for risk automation. The ARES approach enables threat identification utilizing the CSSs, another critical component necessary for modeling threat scenarios [24] that other methodologies like the [36,39] lack. Even though there were efforts like [33] that exploit the CSSs, they did not focus on risk assessment. Those that focus on risk assessment such as [35], assess risk from a vulnerability perspective without considering threats (i.e., CAPEC). Nevertheless, threats must also be identified and be taken into consideration when the risk is estimated [24]; otherwise, the risk will be overestimated. In contrast, ARES considers both the CVEs, in particular, their CVSS score and the CAPECs to estimate risk. Finally, there are efforts like [36], which estimate risk based on best practices and whether they are applied to the CPEs. In this way, however, risk cannot reflect the current threat landscape that the CVEs of the NVD or other open sources communities offer.

## 8. Conclusions

In this paper, we proposed an approach for automated risk estimation in smart sensors environments. The proposed approach is called ARES, complies with NIST’s guidance for conducting risk assessment and estimates risk at the information systems tier. To enable automation, ARES integrates with the BPM LCM, and utilizes the MITRE’s CSSs, FIRST’s scoring system for vulnerabilities and the BPCCS. The latter relies on BPM LCM and allows for defining the BPC. ARES was demonstrated utilizing BPCCS in an application example of a microSCADA controller. Finally, ARES was evaluated, and its applicability was validated utilizing the BPCCS. The limitations of the ARES approach were documented and presented. Towards overcoming the limitations, a new biclustering-based procedure for mapping attack patterns to platforms was proposed.

The implementation of ARES approach utilizing the BPCCS supports integrability, interoperability, composability and security in the Industry 4.0 era. In addition, ARES can help organizations to identify the assets operating inside the business process along with their associated risks. In this way, organizations can conduct risk assessment consistent with their business needs and protect themselves against threats. Another significant merit of ARES is that it leads to increased digital forensics and incident response readiness since all threats to assets are identified in advance. If an attack occurs, digital forensics and incident response teams will be able to know which assets to focus on based on the threats linked to the attack. Furthermore, ARES can contribute to an implementation of the Cybersecurity Framework’s “Risk Assessment” category (ID.RA) of cybersecurity outcomes.

## Figures and Tables

**Table 1:** Risk model of ARES.

	Impact	None	Very Low	Low	Medium	High	Very High
Likelihood							
**Low**		None	Low	Low	Medium	Medium	High
**Medium**		None	Low	Medium	Medium	High	High
**High**		None	Low	Medium	High	High	Very High

**Table 2:** Threat risk factor on a CIA attribute.

	CAPEC	-	Very Low	Low	Medium	High	Very High
CVSS							
-		[Parent CAPEC]	Very Low	Low	Medium	High	Very High
**None**		None	None	None	None	None	None
**Low**		Low	Very Low	Low	Low	Low	Low
**High**		High	Very Low	Low	Medium	High	High

**Table 3:** Threat Identification for “cpe:2.3:a:abb:microscada_pro_sys600:9.3: *:*:*:*:*:*:*.

CVE	CWE	CAPEC
2019-5620	306	12, 36, 62, 166

**Table 4:** Risk estimation for “cpe:2.3:a:abb:microscada_pro_sys600:9.3:*:*:*:*:*:*:* ”.

CVE	CAPEC-36 Impact on			CAPEC-36 Likelihood	Risk on		
	C	I	A		C	I	A
**2019-5620**	High	High	High	Medium	High	High	High
